# 3D Printed Piezoelectric BaTiO_3_/Polyhydroxybutyrate Nanocomposite Scaffolds for Bone Tissue Engineering

**DOI:** 10.3390/bioengineering11020193

**Published:** 2024-02-17

**Authors:** Giovanna Strangis, Massimiliano Labardi, Giuseppe Gallone, Mario Milazzo, Simone Capaccioli, Francesca Forli, Patrizia Cinelli, Stefano Berrettini, Maurizia Seggiani, Serena Danti, Paolo Parchi

**Affiliations:** 1 Department of Civil and Industrial Engineering, University of Pisa, Largo L. Lazzarino 2, 56122 Pisa, Italymario.milazzo@unipi.it (M.M.); patrizia.cinelli@unipi.it (P.C.); maurizia.seggiani@unipi.it (M.S.); 2 Institute for Chemical and Physical Processes (IPCF), National Research Council (CNR), Pisa Research Area, Via Moruzzi 1, 56124 Pisa, Italy; labardi@df.unipi.it (M.L.);; 3 Department of Physics “Enrico Fermi”, University of Pisa, Largo Pontecorvo 3, 56127 Pisa, Italy; 4 Department of Surgical, Medical, Molecular Pathology and Emergency Medicine, University of Pisa, 56126 Pisa, Italy; 5 Department of Translational Research and New Technologies in Medicine and Surgery, University of Pisa, 56126 Pisa, Italy; paolo.parchi@unipi.it

**Keywords:** 3D printing, mechanical properties, piezoelectric coefficients, biodegradation

## Abstract

Bone defects are a significant health problem worldwide. Novel treatment approaches in the tissue engineering field rely on the use of biomaterial scaffolds to stimulate and guide the regeneration of damaged tissue that cannot repair or regrow spontaneously. This work aimed at developing and characterizing new piezoelectric scaffolds to provide electric bio-signals naturally present in bone and vascular tissues. Mixing and extrusion were used to obtain nanocomposites made of polyhydroxybutyrate (PHB) as a matrix and barium titanate (BaTiO_3_) nanoparticles as a filler, at BaTiO_3_/PHB compositions of 5/95, 10/90, 15/85 and 20/80 (w/w%). The morphological, thermal, mechanical and piezoelectric properties of the nanocomposites were studied. Scanning electron microscopy analysis showed good nanoparticle dispersion within the polymer matrix. Considerable increases in the Young’s modulus, compressive strength and the piezoelectric coefficient *d*_31_ were observed with increasing BaTiO_3_ content, with *d*_31_ = 37 pm/V in 20/80 (w/w%) BaTiO_3_/PHB. 3D printing was used to produce porous cubic-shaped scaffolds using a 90° lay-down pattern, with pore size ranging in 0.60–0.77 mm and good mechanical stability. Biodegradation tests conducted for 8 weeks in saline solution at 37 °C showed low mass loss (∼4%) for 3D printed scaffolds. The results obtained in terms of piezoelectric, mechanical and chemical properties of the nanocomposite provide a new promising strategy for vascularized bone tissue engineering.

## 1. Introduction

Bone possesses an intrinsic regenerative capacity by orchestrating a series of biological events involving diverse cell types and intracellular and extracellular molecular signaling pathways, which are ultimately aimed at optimizing its self-repair process and restoring its function [1]. However, there are cases in which bone healing is impaired, such as in bone non-unions and other disease conditions in orthopedic, otologic, oral and maxillofacial surgery [2,3,4]. Bone needs to be replaced in a substantial quantity in the reconstructive surgery of large bone defects, in which the regenerative process is compromised by trauma, infection, tumor resection, skeletal abnormalities, avascular necrosis and osteoporosis [5]. To treat such complex clinical situations, some surgical methods are available; however, these may not be fully resolutive [6]. Bone grafting is a commonly performed surgical procedure conducted to induce bone regeneration in a variety of orthopedic, otologic and maxillofacial procedures; however, autografts, allografts and xenografts show inherent limitations, such as donor site morbidity reduced availability and absorption [7,8,9]. 

Bone substitutes have thus been developed as alternatives to autologous or allogeneic tissue grafts. Among them, much scientific attention has been given to scaffolds made of synthetic and/or natural biomaterials that aim to sustain the growth and the differentiation of bone cells to ultimately induce bone regeneration [10,11]. A wide range of biomaterials are currently investigated as tissue-engineered scaffolds, including biomolecules, ceramics, polymers and their composites [4,11,12,13]. In bone tissue engineering, the search for the optimal biomaterial to fabricate suitable scaffolds for functional bone regeneration is still a subject of study. Ideally, the biomaterial should match the mechanical properties of bone, be easily moldable or printable in precise porous three-dimensional (3D) forms and entail specific signals necessary to stimulate new bone formation [14]. However, *in vivo* survival of *in vitro*-regenerated bone hugely depends on the prompt formation of a vasculature that feeds bone cells, which is difficult to achieve and strongly limits the clinical relevance of tissue engineering strategies [15].

Recently, new insights into the piezoelectric properties of the main bone tissue components, hydroxyapatite and collagen, the latter also widely present in vascular tissues, have attracted the interest of tissue engineers as a possible route to induce the regeneration of vascularized bone [16]. Piezoelectric materials are a class of dielectric materials that can be electrically polarized by the application of mechanical stress [17]. By using piezoelectric materials, such as piezoelectric polymers, ceramics or polymer/ceramic composites, it is possible to fabricate scaffolds that promote the growth and differentiation of bone cells and small vessels [18,19]. Indeed, the electrical signals resulting from mechanically stimulated piezoelectric materials have revealed ability to regenerate and repair tissues through definite pathways [20]. The piezoelectric properties of polymers are generally lower than those of piezoelectric inorganic crystals, but polymers possess the great advantage of processing flexibility via different manufacturing methods. Mechanically, polymers have high fracture toughness and high impact resistance as compared to inorganic materials, which are hard and fragile. The best piezoelectric polymer class is led by poly(vinylidene fluoride) (PVDF) and its copolymers, with a piezoelectric coefficient in the order of 20 pC/N [21]. As a fluoropolymer, PVDF is thermally and chemically stable and thus non-biodegradable, and so it may activate a fibrotic capsule formation as a drawback [22]. However, due to its remarkable piezoelectric properties, PVDF is currently studied for engineering mechanically responsive tissues, such as bone and lung [18,23].

Among the polymers endowed with an appreciable piezoelectric response, which are bioresorbable in the human body, the polyhydroxyalkanoate (PHA) family represents an emerging class [24]. The piezoelectric effect of these polymers originates primarily from their crystalline properties. When an external stress is applied, the internal rotation of molecular dipoles in the crystalline phase gives rise to spontaneous electrical polarization. Polyhydroxybutyrate (PHB) is a PHA family member reported to have a piezoelectric coefficient of about 1.3 pC/N [25], whereas PHB-based scaffolds are reported to have a piezoelectric coefficient of about 3 pC/N [26]. For this reason, various studies have promoted the use of PHB and its copolymers, among other piezoelectric materials, to fabricate scaffolds for bone regeneration [27]. Piezoelectric ceramics with a perovskite structure generally exhibit a larger piezoelectric effect compared to other types of materials [28,29]. Characterized by a high dielectric constant, barium titanate (BaTiO_3_) shows a *d_33_* coefficient of 191 pC/N [30,31]. Importantly, it has been reported that the piezoelectric property of BaTiO_3_ has also a positive influence on cellular proliferation [32]. 

In this study, we developed and characterized BaTiO_3_/PHB nanocomposites to produce biodegradable piezoelectric scaffolds for bone tissue engineering via 3D printing. The thermal, mechanical, morphological and piezoelectric properties, as well as the printability of the nanocomposites produced with different BaTiO_3_/PHB weight ratios, were investigated. Finally, mechanical tests and preliminary *in vitro* biodegradation studies on the 3D printed scaffolds were performed. Making piezoelectric yet biodegradable scaffolds available could open avenues for functional bone replacements in orthopedic, otologic and maxillofacial translational research. 

## 2. Materials and Methods

### 2.1. Materials

PHB #P226 was purchased from Biomer^®^ (Krailling, Germany). As declared by the supplier, it has the following properties: density = 1.25 g/cm^3^, purity = 98% ± 2%, melt flow index = 9–13 g/10 min at 180 °C, crystallinity = 60%, melting temperature = 170 °C, maximum degradation temperature = 284 °C. For this product, M_w_ = 611 kg/mol and polydispersity index = 3.1 are reported in the literature [33]. Barium titanate powder (purity 99%, particle size < 3.0 µm as declared by the supplier, density = 6.02 g/cm^3^) was supplied by Sigma-Aldrich (Steinheim, Germany).

### 2.2. Nanocomposite Preparation

PHB pellets were dried in a Binder Oven 741 L-FD 720 (Binder GmbH, Tuttlingen, Germany) for 24 h at 60 °C to remove residual humidity. Then, four nanocomposites, obtained by adding PHB with 5%, 10%, 15% and 20% w% BaTiO_3_, were prepared (Table 1).

The nanocomposites were obtained by mixing PHB pellets with appropriate quantities of BaTiO_3_ powder in a blade mixer, then extruded and granulated. In detail, 100 g of BaTiO_3_ were added to 1.9 kg of PHB, thus obtaining the first nanocomposite pellets, namely, 5/95 (w/w%) BaTiO_3_/PHB. Therefore, 400 g were extruded to produce a 5/95 (w/w%) BaTiO_3_/PHB filament suitable for 3D printing. The residual 5/95 (w/w%) nanocomposite (i.e., 1.6 kg) was added with 89 g of BaTiO_3_ powder to obtain BaTiO_3_/PHB 10/90 (w/w%) nanocomposite pellets, i.e., containing 10% (w%) of BaTiO_3_, and 400 g of these pellets were extruded to obtain the second filament. In the same way, BaTiO_3_/PHB 15/85 (w/w%) and BaTiO_3_/PHB 20/80 (w/w%) were obtained, both in the form of pellets and filaments.

The production of each nanocomposite filament was performed in a Brabender Extruder GmbH & Co. KG (Duisburg, Germany), with a maximum screw diameter of 30 mm, minimum diameter of 19 mm and maximum capacity of 15 kg/h, namely, suitable for loading the filler into the polymer matrix with an improved dispersion of nanoparticles. During the extrusion of nanocomposites in the form of pellets, different temperature profiles in the extruder zones, from 175 °C up to 185 °C at the extruder nozzle, and a screw speed of 80 rpm were used. Torque value provided an evaluation of the polymer fluidity at the time of melting in the Brabender mixer (by WinMix program). The torque is an index of the polymer fluidity and processability that is related to the viscosity of the material inside the instrument. The extruded strands were cooled in a water bath at room temperature and cut into pellets with an automatic knife cutter. All pellets were finally dried in an oven at 60 °C. Therefore, to obtain a suitable diameter of the filament (i.e., 1.75–1.80 mm), a rotating coil at the extruder outlet was used. The filaments were utilized for 3D printing, while the corresponding pellets for the other characterization techniques.

### 2.3. Preparation of Samples for Analyses

After the extrusion and the filament granulation, all the obtained pellets were dried for 24 h in a Binder Oven 741 L-FD 720 (Binder, GmbH, Tuttlinger, Germany) at 60 °C and further enclosed in vacuum bags to eliminate traces of humidity before processing by an Injection Molding Press Megatech H10/18–1 (TECNICA DUEBI S.r.l., Fabriano, Italy). Injection molding was used to prepare dog-bone samples for mechanical characterization under tensile mode. The injection molding parameters, related to the different samples shaped according to ISO 527-1A (i.e., 150 mm length, 20 mm width, 5 mm thickness), were: (i) Operative temperatures into the screws: 180/180/178 °C; (ii) Temperature of the mold: 60 °C; (iii) Dosing: 48 mm; (iv) Speed: 70%; (v) Injection pressure: 80 bar; (vi) Cooling time in the mold: 10 s. The same parameters were used for both plain PHB and the nanocomposites with different weight fractions of barium titanate. 

To obtain the films used for piezoelectric analysis, about 2.5 g of the different materials were placed between Teflon square sheets and compression-molded with a top plug between two heated steel plates (mold cavity) in a NOSELAB ATS Manual Laboratory Heat Press (Nova Milanese, Italy) at 180 °C with a pressure of 4 metric tons for 4 min to allow the material to contact all areas of the mold. After production, each film was rapidly removed from the press and detached from the Teflon sheets.

### 2.4. Thermal and Thermogravimetric Analyses

Differential Scanning Calorimetry (DSC) was employed to investigate the thermal properties of the produced samples, using a Q200 differential scanning calorimeter (TA-Instrument, Midland, ON, Canada). Nitrogen, set at 50 mL/min, was used as purge gas for all measurements. The materials used for DSC analysis were cut from the samples obtained after the mixing and extrusion process. Aluminum pans were loaded with amounts of samples ranging in 10–15 mg and sealed before measurements. The method set-up to perform all thermal analyses was based on a sequence of heating–cooling–heating ramps, corresponding to the time–temperature profile reported in Figure S1. Melting temperature (*T_m_*) and crystallization temperature (*T_c_*) of the samples were evaluated in correspondence with the melting peak and the crystallization peak, respectively. The enthalpies of melting (Δ*h_m_*) were determined from the areas under the corresponding peaks in the heating thermograms. The degree of crystallinity (*X_c_*) was calculated from the heating scans of PHB, as follows (Equation (1)):

(1) $$X_{c-heat\;}\left(\%\right)=\left[\frac{{∆h}_{m}}{{∆h}_{m}^{0}}\right]\;\cdot \;100$$

where ${∆h}_{m}\;$is the melting enthalpy of samples and ${∆h}_{m}^{0}$ is the melting enthalpy of 100% crystalline polymer, which in this work was calculated to be 146 J/g for PHB through the relationship in Equation (2) [34,35]:

(2) $${∆h}_{m}^{0}\;\left(T\right)=80.44+0.492\;‧\;T-0.0007\;‧\;T^{2}$$

with *T* in °C.

For the nanocomposites, the formula of the crystallinity degree is given by Equation (3):

(3) $$X_{c-heat}\left(\%\right)=\left[\frac{{∆h}_{m}}{{∆h}_{m}^{0}\left(1-w_{Filler}\right)}\right]\;·\;100$$

in which $w_{Filler}$ is the weight fraction of BaTiO_3_ in the nanocomposite. Finally, the crystallinity degree that develops during the cooling ramp was calculated using Equation (4):

(4) $$X_{c-cool}\left(\%\right)=\left[\frac{{∆h}_{c}}{{∆h}_{c}^{0}\left(1-w_{Filler}\right)}\right]\;·\;100$$

where ${∆h}_{cc}$ is the measured heat of cold crystallization between approximately 70 °C and 120 °C, and ${∆h}_{c}^{0}$ is the heat of crystallization calculated as in Equation (2).

Dynamic mechanic thermal analysis (DMTA) was used to determine the glass transition temperature (*T_g_*) and was carried out on a Gabo Eplexor^®^ (Selb, Germany) with a 100 N load cell. Test bars (size: 2.0 mm × 5.0 mm × 1.5 mm) were cut from films previously prepared with the compression molding, through the Elastocon cutting dies. The temperature range was set from −50 °C to +50 °C. Thermogravimetric analysis (TGA) was performed on samples in the form of pellets using a TA Q-500 (TA Instrument, Waters LLC, New Castle, DE, USA). An amount of material, which, depending on the sample, varied between 12 mg and 30 mg, was loaded into a platinum pan and heated from room temperature to 600 °C at 10 °C/min under a nitrogen atmosphere. TGA was used to evaluate the thermal stability of different samples in view of their subsequent processing by melting extrusion and 3D printing.

### 2.5. Piezoelectric Characterization of Nanocomposites

The piezoelectric study was conducted on films by improving a custom-made experimental setup named “piezo-gauge” [36]. Samples of 3.8 cm × 1 cm with a thickness of ∼100 μm were cut and suitably prepared by applying 0.5 cm × 1 cm sized Teflon clamps at their edges. Briefly, the sample was fixed to a holder, one end was attached to a harmonic steel cantilever-shaped spring, and the other end of the sample was attached to a to a software-controlled slide, which allowed adjustment and stabilization of the mechanical tension of the sample. The controller slide was set to keep the sample under a constant stress condition during the measurements, to consider and compensate for any mechanical relaxation over the measurement time. 

An electric potential was applied in the transverse direction (*z*-axis, across the sample thickness) through a pair of planar electrodes, positioned as close as possible to the sample without touching it. The distance between the slabs was adjusted by microtranslators. The transverse electric field produced a longitudinal deformation (along the *x*-axis, along the sample length direction) proportional to the transverse, converse piezoelectric coefficient *d*_31_ of the material. Alternating electrical drive at the resonance frequency of the measurement cantilever spring produced an enhancement of its vibration due to the harmonic oscillator quality factor Q of the cantilever, which could be measured by recording its resonance curve. Finally, bending of the steel cantilever was detected by an optical lever method, where a laser beam was reflected by a mirror attached on the spring and its deflection was detected by a four-quadrant split photodiode. After placing the sample, the setup was enclosed into a box to isolate it from acoustic noise sources. The measurement started with the acquisition of the resonance signal by sweeping the AC voltage frequency applied to the electrodes positioned to face to the sample. 

The acquisition of the spectra, for each sample, was carried out by means of an acquisition software (in LabView*^®^* v. 2018) created specifically for these measurements. The acquired spectra were analyzed by means of the software Origin*^®^* (v. 2018) through a Lorentzian Fit to obtain the parameters of interest, namely resonance frequency (Hz), resonance width (Hz) and maximum amplitude *V_RMS_* (mV). The converse piezoelectric coefficient *d*_31_ was calculated according to Equation (5):

(5) $$d_{31}=C\;\frac{\varepsilon_{s\;}V_{RMS}}{F\;V_{exc}}$$

where *V_exc_* is the amplitude of the applied potential; *C* is a calibration constant that depends on spacing between plates [36]; and $\varepsilon_{s}$ is the apparent dielectric constant of the sample placed in the gap between the electrodes, resulting from Equation (6):

(6) $$\varepsilon_{s}=\varepsilon_{d}-\frac{\eta_{d}}{\eta }\left(\varepsilon_{d}-1\right)$$

in which $\eta_{d}$ is the sample thickness, *η* is 2 mm and $\varepsilon_{d}$ is the effective dielectric constant of the sample. The dielectric constant $\varepsilon_{d}$ was separately measured by dielectric spectroscopy (DS) on disk-shaped specimens (~100 µm thick) with a diameter of 15 mm, whose circular surfaces had preliminarily been sputtered with platinum in order to apply a conductive coating (~15 nm thick). DS was carried out by collecting the complex admittance spectra of a plain circular capacitor cell containing the specimen, which was connected to an impedance analyzer (model 4294A from Agilent, Santa Clara, CA, USA). Calibration of both the circuit set-up and the cell was obtained by measuring specimens of non-polar standard materials (i.e., PTFE, disc and air gap). The dielectric spectra for each tested material were thus obtained in a 60 Hz–10 MHz frequency range with simple calculations that considered the exact dimensions of the specimens. The value of $\varepsilon_{d}$ for each sample was then determined as the real part of the complex dielectric permittivity at the frequency of interest, i.e., 300 Hz, for the analysis of piezoelectric characterization data. The “F” factor in Equation (5) is of the order of the quality factor Q of the measurement spring. Polytetrafluoroethylene (PTFE) and poled PVDF films were used as negative and positive controls, respectively.

### 2.6. 3D Printing of Nanocomposites

A pore size of 1 mm was selected for this research. After the selection of modeling parameters, the unit cell was modeled and patterned in three directions (*x*, *y*, *z*) to obtain scaffolds with size 10 × 10 × 10 mm^3^. The dimensions of the sample were selected in accordance to the standard ISO 13314:2011, which requires the length of each side not to be lower than 10 mm. For CAD modeling, porosity was calculated as a volumetric porosity, using Equation (7) [37]:

(7) $$Porosity\;(\%)=\left(1-\frac{V}{V_{s}}\right)\times 100$$

where V and V_s_ are the volume of porous scaffold and the solid structure, respectively. The volume of porous samples was derived by the CAD software (Solidworks*^®^* v. 2021) and used to determine the porosity. Samples were fabricated using the instrument Creality 3D-CR-10S5 (Shenzhen Creality 3D Technology Co., Ltd., Shenzhen, China), with a nozzle diameter of 0.4 mm. The scaffolds were fabricated using a 90° lay-down pattern with a continuous contour filament to achieve an interconnected porous reticular structure. Temperature and injection speed were changed during the printing process to obtain scaffolds with a pore size of 1 mm. The designs were exported in a rapid prototyping format (.stl file format) and uploaded to the 3D printing software (Slic3r v. 1.2.9), in which the model was sliced by a slicing algorithm. The 2D sliced layers were then built by the printer layer by layer until the final object was physically formed. For each extruded nanocomposite, filaments with diameters ranging in 1.75–1.80 mm were used to print the scaffolds. The printing parameters were as follows: Layer height: 0.2 mm; Infill: 100%; Printing temperature: 190 °C; Build plate temperature: 50 °C; Print speed: 50 mm/s; Build plate adhesion brim: 8 mm.

### 2.7. Tensile and Compression Tests

The mechanical analysis of the nanocomposites was performed on classical dog-bone specimens for tensile tests, and on cube-shaped specimens for compression tests, the latter obtained by blade cutting of square-section dog-bones. In particular, ordinary quasi-static tests were carried out at 10 mm/min for the tensile test, and at 1 mm/min for the compression test, by means of the MTS Criterion model 43 universal tensile testing machine (Eden Prairie, MN, USA) equipped with a 10 kN load cell and interfaced with a computer running MTS Elite Software (MTS Testsuite version 4.1). The tests were performed 48 h after injection molding of the samples. Five specimens (*n* = 5) were tested for each sample, according to the ASTM D 638, to evaluate the average value of the Young’s modulus, maximum tensile stress, elongation at break and maximum compressive strength.

### 2.8. Morphological and Porosity Analysis

The surface morphology and elemental analysis (EDX) of the nanocomposites were performed via scanning electron microscopy (SEM) using the FEI ESEM Quanta 450 FEG instrument (Waltham, MA, USA). Samples were mounted with on aluminum stubs, fixed with carbon tape and sputtered with platinum (Leica EM ACE600). BaTiO_3_ nanoparticle size at the composite surface was evaluated using ImageJ software (v. 1.53t, Wayne Rasband National Institutes of Health, Bethesda, MD, USA) on SEM micrographs (*n* = 100). The porosity of printed samples was determined using Equation (8), as a gravimetric porosity [38]:

(8) $$Porosity\;(\%)=\left(1-\frac{\rho_{sc}}{\rho_{m}}\right)\;·\;100$$

where ρ*_sc_* is the apparent density of the scaffold (calculated as the ratio between the scaffold weight and its volume) and ρ*_m_* is the bulk density of the material. The scaffold volume was obtained by measuring length, width and height of the sample. Finally, the pore size was calculated using the ImageJ software on scaffold micrographs taken with a Wild M3 Heerbrugg Optical Microscope (Wild Heerbrugg, Switzerland) equipped with a dark-field base and 6.4×, 16.0× and 40.0× magnification objectives. 

### 2.9. Biodegradation Tests

BaTiO_3_/PHB 0/100 and 20/80 (w/w%) scaffolds (n = 3 for each species), having initial average weights of 331 mg and 436 mg, respectively, were placed in 8 mL of saline solution (pH 7.4, 0.9% NaCl) and kept in an oven at 37 °C. Samples were periodically removed from the solution, washed in distilled water and dried in an oven for 3 h at 60 °C. One sample for each type was observed after 6 weeks via SEM. Biodegradation was assessed biweekly by measuring the weight loss until 8 weeks using a laboratory scale. 

### 2.10. Statistical Analysis

Statistical analysis was carried out using independent Student’s *t*-test and one-way ANOVA, setting a significance probability threshold (*p*) of 0.05. All mechanical data were represented as mean ± standard deviation.

## 3. Results

### 3.1. Production of the Nanocomposites

**Figure 1:**
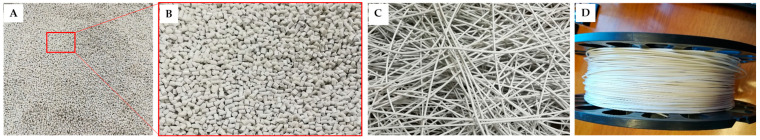
Photographs showing BaTiO_3_/PHB 5/95 (w/w%) nanocomposite production: (**A**,**B**) pellets and (**C**,**D**) extruded filament.

The nanocomposites were obtained by mixing PHB pellets with different contents of BaTiO_3_ powder in a blade mixer, then extruding and granulating them (Figure 1A,B). After extrusion using a screw speed at 80 rpm and a temperature in the 175–185 °C range, filaments were obtained with diameters ranging in 1.75–1.80 mm (Figure 1C,D). 

The torque values showed that, after filler addition, the viscosity first increased and then decreased with time down to a value of approximately 0.6 N‧m, which is very close to that of plain PHB (Figure S2). 

### 3.2. Characterization of the Nanocomposites

**Figure 2:**
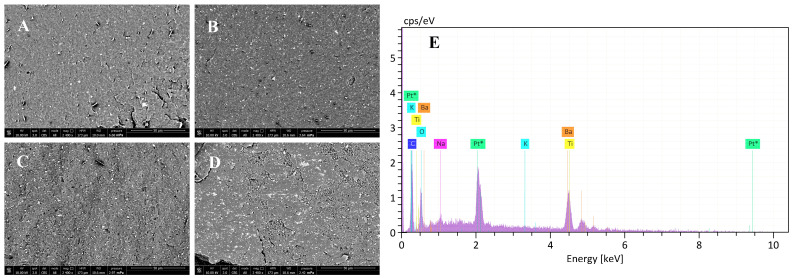
Results of morphological analyses on the BaTiO_3_/PHB nanocomposites. (**A**–**D**) Representative SEM micrographs for (**A**) 5/95, (**B**) 10/90, (**C**) 15/85 and (**D**) 20/80 (w/w%) formulations; voltage is 10 kV, magnification is 2400×, scale bar is 50 µm. (**E**) Representative spectrum of EDX analysis showing the presence of Ba and Ti at the material surface; * indicates the element (i.e., Pt) added for sputter coating the surface.

After production, SEM observations showed that a homogeneous dispersion of the BaTiO_3_ particles in the PHB matrix was achieved for all compositions (Figure 2A–D). The multiple extrusion process enabled the filler dispersion within the polymer matrix, and only low amounts of agglomerates, less than 0.3% for each sample, were counted on SEM micrographs. Most (i.e., 75–78%) nanoparticles visible at the surface had diameters ranging in 40–100 nm, and only 0.03% had size ≥ 1 μm; this result is in agreement with SEM analysis carried out on a sample of BaTiO_3_ particles as supplied (Figure S3). The presence of Ba and Ti at the nanocomposite surface was confirmed by EDX analysis (Figure 2E). In some EDS analyses, the presence of sodium, magnesium, chlorine and potassium was revealed (Figure S4). 

The DSC analysis performed on the composites obtained after mixing showed that the filler addition did neither significantly affect the melting temperature, nor the crystallization temperature of PHB as measured during the first and the second ramps. Instead some differences arose in the melting process profiles observed during the third ramp, with *T*_m_ decreasing as the filler content was increased. Additionally, the nanocomposites showed a crystallinity degree lower than that of plain PHB “as loaded” into the DSC instrument (first ramp), after being re-cooled down from the melt (second ramp) and re-heated (third ramp). Thus, the presence of the filler could be deemed to interfere, although only slightly, with the crystallization process (Table 2). 

The DSC thermograms also showed the presence of a small endothermic peak at about 50 °C, which was also observed in the cooling scan at 20 °C (Figure S5).

**Figure 3:**
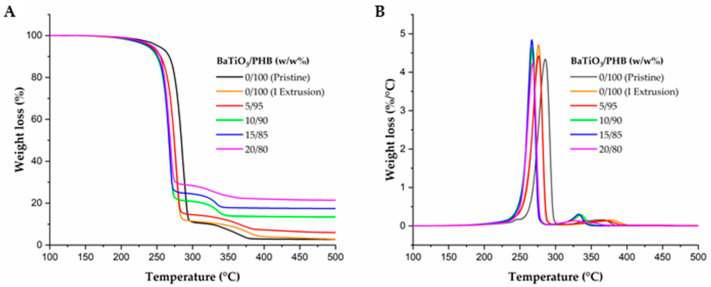
Results of TGA showing the stability of all the produced BaTiO_3_/PHB nanocomposites and plain PHB until reaching 200 °C: (**A**) integral, and (**B**) derivative weight fraction losses, as functions of temperature.

The TGA, reported in Figure 3, revealed that a two-step degradation process occurred in all samples, mainly in a temperature range of 200–400 °C, above which the degradation of the PHB matrix was almost complete (~98%). The differences observed in the residuals above 400 °C reflected the growing content of the ceramic filler, which had higher thermal stability than the polymeric matrix and was not degraded in the explored temperature range.

**Figure 4:**
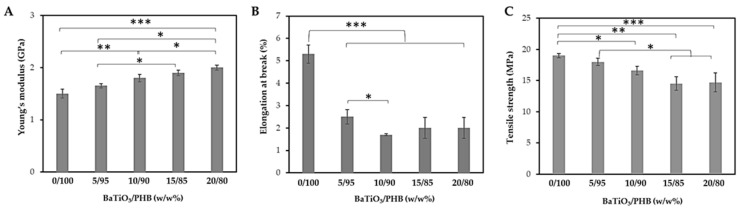
Results of tensile mechanical tests performed on the BaTiO_3_/PHB nanocomposites and plain PHB. (**A**) Young’s modulus, (**B**) Elongation at break, (**C**) tensile strength. Results are reported as mean ± standard deviation; statistical significance: * *p* < 0.05; ** *p* < 0.001; *** *p* < 0.0001.

The outcomes of the tensile tests conducted on dog-bone specimens of both plain PHB and BaTiO_3_/PHB composites are shown in Figure 4. The Young’s modulus values increased with increasing BaTiO_3_ content. 

The higher stiffness given by the higher weight fraction of the filler was accompanied by a progressive reduction of the elongation at break, which dropped from 5.3% down to 2.5–1.7%, rendering the composites more brittle than the plain PHB. Similarly, the tensile strength was affected, even if less significantly, by increasing the filler content up to 20% (w/w%). Preliminary compression tests performed on cubic specimens of plain PHB to determine the effect of the extrusion cycles showed that this property was reduced upon multiple melting processes (Table 3). 

**Figure 5:**
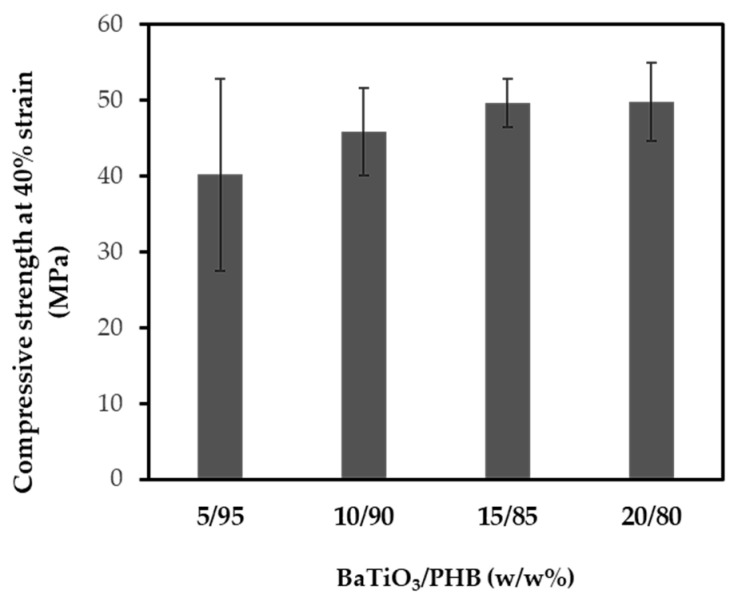
Results of compressive mechanical tests performed on the BaTiO_3_/PHB nanocomposites at 40% strain.

The results of the compressive tests subsequently performed on the nanocomposites indicated that the presence of the filler averagely raised the compressive strength at 40% strain, although such an effect was not statistically significant (*p* = n.s.) (Figure 5). However, it is worth noting that these values were always higher than those of the plain PHB after the corresponding extrusion cycles.

**Figure 6:**
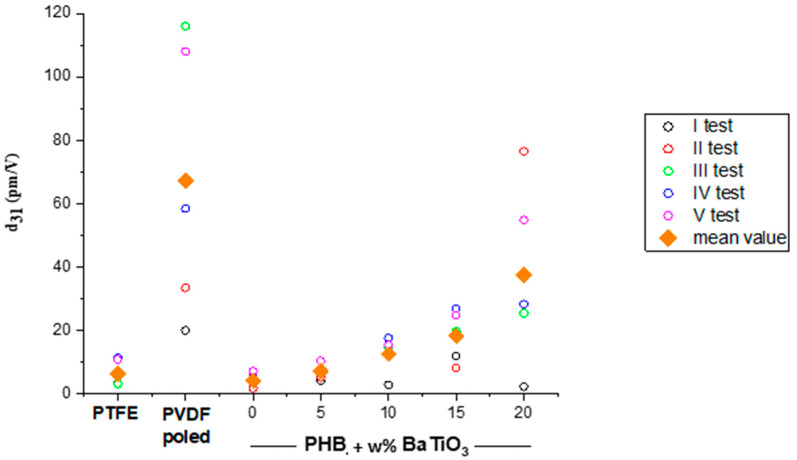
Graph showing the converse piezoelectric coefficient *d*_31_ results obtained for PHB and BaTiO_3_/PHB nanocomposites. PTFE and poled PVDF films were used as negative and positive controls, respectively.

The compression molded films showed a thickness in the 100–120 µm range. Several tests were performed to obtain the converse piezoelectric coefficient *d*_31_ (pm/V) of the samples, which increased by nearly 10 times when 20% w/w% BaTiO_3_ was added to the plain PHB (Figure 6). Table 4 reports the values of the dielectric constant *ε_d_*, the converse *d*_31_ and the direct *g*_31_ piezoelectric coefficients for plain PHB and BaTiO_3_/PHB nanocomposites.

### 3.3. Production of the Nanocomposite Scaffolds

**Figure 7:**
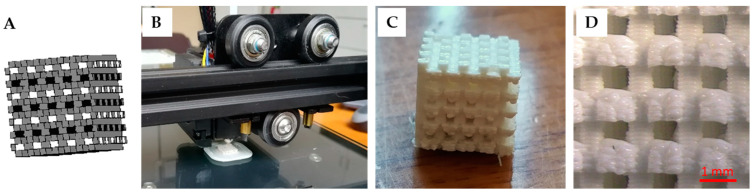
3D printing process and resulting BaTiO_3_/PHB nanocomposite structures: (**A**) CAD model; (**B**) Photograph showing the 3D printing operation; (**C**) Representative photograph of 3D printed 5/95 (w/w%) BaTiO_3_/PHB scaffold; (**D**) Pore and surface features of the 3D printed 5/95 (w/w%) BaTiO_3_/PHB scaffold obtained via stereomicroscopy.

The PHB/BaTiO_3_ filaments resulted in the fabrication of interconnected, porous cubic scaffolds with large pore size (Figure 7). Scaffolds with an alternated filling pattern of 0°–90° layers were printed with a nozzle diameter of 0.4 mm, a printing speed of 50 mm/s and an extrusion temperature of 190 °C. The extruded patterns solidified quickly due to the heat dissipation at room temperature, hence enabling a layer-by-layer deposition.

The behavior of the different nanocomposites during the printing process was similar. Upon 3D printing, the 20/80 (w/w%) BaTiO_3_/PHB filament resulted more brittle than the 0–15% (w/w%) BaTiO_3_/PHB counterparts; however, by slightly adjusting the temperature and printing speed parameters, it was still possible to print it successfully. Figure 7D shows a representative stereomicroscopy image of 3D printed 5/95 (w/w%) BaTiO_3_/PHB scaffold used to evaluate pore size and porosity. 

### 3.4. Characterization of the Nanocomposite Scaffolds

The pore size and the porosity values determined experimentally in the plain PHB scaffolds, in the lowest (5 w%) and the highest (20 w%) BaTiO_3_-content scaffolds resulted always lower than those predicted by the CAD model, as shown in Table 5. Specifically, the experimental pore size ranged in 0.60–0.77 mm, in place of the designed value of 1.00 mm, and the porosity ranged in 54–62% in place of the designed value of 70%. 

**Figure 8:**
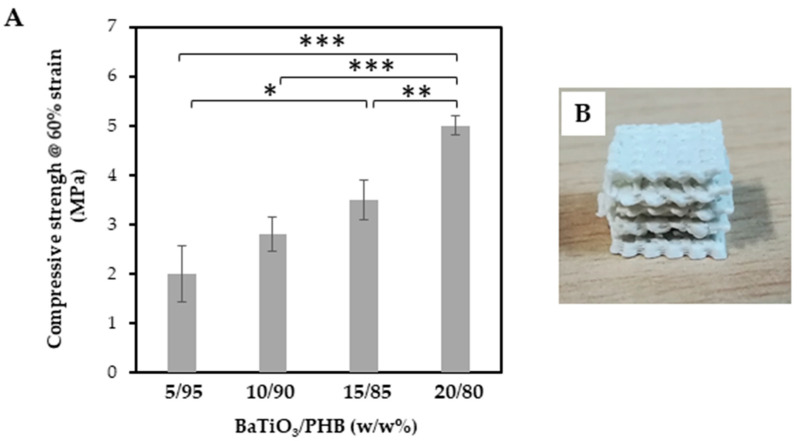
Results of compressive tests performed on the 3D printed BaTiO_3_/PHB nanocomposite scaffolds at 60% strain: (**A**) Compressive strength; (**B**) Representative photograph of a 15/85 (w/w%) BaTiO_3_/PHB scaffold after compression. Results are reported as mean ± standard deviation; statistical significance: * *p* < 0.05; ** *p* < 0.001; *** *p* < 0.0001.

The compressive strength of the 3D printed PHB/BaTiO_3_ scaffolds was derived by using the MTS dynamometer to squeeze the specimens up to 60% strain. The maximum compressive strength of the printed scaffolds varied in a range of 2–5 MPa and increased with increasing the filler content (Figure 8A). The compressed scaffolds did not fracture, but their pore walls collapsed, and the surface pores melted into each other (Figure 8B). 

**Figure 9:**
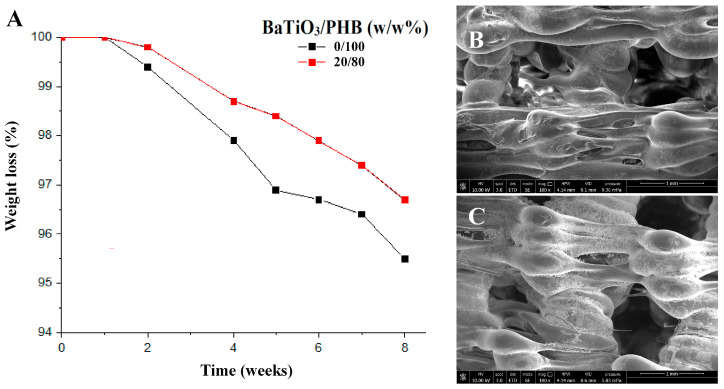
Results of biodegradation test performed on the 3D printed 0/100 (w/w%) and 20/80 (w/w%) BaTiO_3_/PHB scaffolds at 37 °C in saline solution for 2 months: (**A**) Graph showing the average weight loss, obtained via biweekly measurements; (**B**,**C**) SEM analysis after 8 weeks: (**A**) Plain PHB scaffold, and (**B**) 20/80 (w/w%) BaTiO_3_/PHB scaffold. Voltage 10 kV, 100× magnification, scale bare is 1 mm.

The degradation of plain PHB and 20/80 (w/w%) PHB/BaTiO_3_ scaffolds upon immersion in saline solution at 37 °C was studied by performing periodic weight measurements for 8 weeks and by recording the mass loss. At the endpoint, the scaffold morphology was observed via SEM to visualize the degradation effects on the scaffold structure. Figure 9 highlights that both scaffold types slowly biodegraded in 2 months. 

None appreciable weight change was detected after the first week for both samples, and afterwards, weight loss started to occur at a slow rate, with a slightly faster kinetics in plain PHB scaffolds. As an example, 2.1% weight loss was achieved in 4 weeks by the plain PHB scaffold, while the same was reached in 6 weeks by 20/80 (w/w%) BaTiO_3_/PHB scaffolds. At the endpoint, the weight of the PHB scaffold showed an average 4.5% decrease, while the 20/80 (w/w%) BaTiO_3_/PHB scaffold degradation was slower, showing an average 3.3% weight reduction (Figure 9A). 

SEM micrographs allowed the observation of surface morphology and architecture of both scaffolds, which evidenced some initial changes, such as some signs of surface erosion and early disruption of the inner porosity (Figure 9B,C).

## 4. Discussion

The search for biomaterial formulations to fabricate customized scaffolds capable of promoting the regeneration of functional bone tissue *in vivo* under the tissue engineering paradigm is still a challenging matter. It has been postulated that a successful scaffold should act as a temporary extracellular matrix (ECM) by providing not only a congruent mechanical support, but also biophysical and/or biochemical stimuli [39,40]. Mechanotrasduction is one of the main functions of the osteoblasts, which is adjuvated by the bony tissue microenvironment, including ECM signals [41,42,43]. Specifically, it has been shown that the main components of bone ECM, such as collagen fibrils and hydroxyapatite, possess piezoelectric properties, namely, they are able to provide electric signals upon mechanical stress, which ultimately activate signaling pathways in bone cells [44,45]. Piezoelectric properties have also been demonstrated in blood vessels, in which they are primed by elastin and collagen fibrils, thus supporting the hypothesis that piezoelectric scaffolds could enhance both osteogenesis and vasculogenesis *in vivo* [46,47]. In fact, one of the main drawbacks reported after cellularized scaffold implants *in vivo*, especially for the treatment of large defects, was an inefficient blood vessel ingrowth within the new tissue, which led to the death of the implanted cells [15,48]. It was also found that piezoelectric scaffolds could promote the formation of functional and vascularized bone [16]. Finally, since appropriate geometry design and fabrication process are mandatory for a successful scaffold structure, both these stages can greatly benefit from CAD-assisted manufacturing techniques, like 3D printing, which are becoming remarkable tools to address bone defects of specific shapes and sizes, thus enabling personalized medicine approaches [49].

This study was aimed at the fabrication of new stimuli-responsive scaffolds via 3D printing to provide electric bio-signals naturally present in bone tissue by using piezoelectric materials. Specifically, we selected PHB as a rigid and long-term bioresorbable polymer entitled with good piezoelectric properties and excellent biocompatibility, i.e., superior to that of poly(lactic acid), as the local pH is stable during the PHB biodegradation process [50]. We thus combined it with BaTiO_3_ nanoparticles, which are strong piezoelectric nanoceramics, to obtain a set of nanocomposites. The ultimate purpose of this research was to improve the mechanical and piezoelectric properties of plain PHB and to demonstrate the nanocomposite printability into porous scaffolds that could be suitable for bone tissue engineering application. In fact, having a mechanically strong piezoelectric scaffold mainly composed of a biodegradable polymer is thought to enhance new bone formation once implanted *in vivo*, thus overcoming the limits of PVDF-based scaffolds, which have less suited mechanical properties and do not biodegrade [51]. 

PHB belongs to the PHA family and is one of the best performing biopolymers currently studied in the biomedical field [52,53]. Nowadays, the research interest is focusing on this biopolymer class thanks to the possibility of offering both a green alternative and a more sustainable solution to replace products that involve high environmental impacts, along with its excellent biocompatibility and biodegradation properties, which are suited for tissue engineering [54]. PHB-based nanocomposites have been investigated for bone regeneration aimed at achieving desirable mechanical properties, e.g., by using nanoclay as a filler [55]. However, the research on PHB nanocomposites should also pursue further improvement in the mechanical and piezoelectric performance of the final device. In this view, we selected PHB as a polymer matrix, due to its excellent biocompatibility and its good mechanical and piezoelectric properties [24,55], and barium titanate nanoparticles as a filler, due to the high stiffness and excellent piezoelectric properties of this ceramic [56].

This study included four main research phases. In the first phase, BaTiO_3_/PHB nanocomposites were prepared with different filler contents, 5%, 10%, 15% and 20% (w/w%), by processing them in a Brabender mixer at 45 rpm for 900 s at 180 °C. A crucial step was to ensure homogenous nanoparticle dispersion in the polymer matrix, given the high nanoceramic stiffness. Multiple extrusion cycles (up to 4) at a screw speed of 80 rpm and a temperature ranging in 175–185 °C allowed uniform nanoparticle dispersion to be achieved in all the nanocomposites without using any additives or plasticizers, as they could compromise the biocompatibility of the device. Most of the dispersed visible nanoparticles had diameters ranging in 40–100 nm, with only 0.03% particles being larger than 1 μm, thus corroborating the absence of nanoparticle agglomeration in the mixing process. The EDX spectra also showed few impurities, which could either be attributed to the presence of nucleating agents in the polymer matrix, or be remnants from synthesis. The torque–time curves, which provide information on the effectiveness of the mixing, the rheological behavior and the thermal and shear stability, showed that PHB had better thermal stability than its copolymer poly(hydroxybutyrate-co-valerate) (PHBV) at 180 °C, in agreement with other studies [57]. Since it is known that, in addition to thermal stress, the screw speed of the extruder and the related shear deformation can affect the degradation behavior of a polymer, the produced samples were further analyzed by DSC and TGA to assess the extent of this influence [58].

As such, the second phase of this study focused on the thermal, mechanical and piezoelectric characterization of the nanocomposites. The TGA showed that both the pristine polymer and the produced nanocomposites underwent a two-step degradation process from 200 °C to 400 °C. During the first step, occurring between 200 °C and 300 °C, the PHB mass was reduced by ∼90% in all samples; then, the residual PHB was almost consumed in the second step, occurring between 300 °C and 400 °C. Such a degradation pattern is in line with the ones reported by other authors [55,59]. In particular, among the three different PHB types studied by Pradhan et al., the behavior of our samples was very similar to that of the sample biosynthesized by *Bacillus megaterium* [59]. By comparing the curves of the pristine PHB matrix with that of PHB after one extrusion cycle and that of 5/95 (w/w%) BaTiO_3_/PHB nanocomposite, which also had one extrusion cycle, it is possible to infer that extrusion affected (i.e., slightly decreased) the thermal stability of the matrix more than the mere addition of the ceramic filler. In this light, the trend observed for the main degradation temperatures in the nanocomposites with BaTiO_3_ content higher than 5% (w/w%), which apparently decreased slightly with filler content, could be a consequence of the increasing number of extrusion cycles required to process the formulations, rather than the further additions of our ceramic filler. However, after the first extrusion cycle, the thermal stability settled at a limiting value for 10/90, 15/85 and 20/80 (w/w%) BaTiO_3_/PHB nanocomposites. The slight influence of the filler content on the thermal stability of the matrix is in line with findings reported elsewhere on PHB/montmorillonite nanocomposites [55]. 

The results obtained from DSC showed that, up to the melting process, the thermal properties of the nanocomposites slightly changed with respect to those of PHB. In fact, both *T_m_* and *T_c_* were nearly unaffected, while the crystalline fraction progressively, although slightly, deteriorated with increasing filler content, which corresponds to increasing extrusion cycles. A slight decrease in *T_g_* as measured by DMTA was observed in samples with increasing filler content, but even this variation occurred only to the point of a limiting inferior value. Such behavior is similar to that observed for the main degradation temperature obtained by TGA, and thus, both the DMTA and TGA outcomes suggested that the thermo-mechanical action exerted by each successive extrusion cycle may have caused a progressive slight reduction in the average molar mass of the PHB molecular chains, although to a limited extent. The literature reports modifications of the melting behavior of semi-crystalline polymers due to filler addition [60]. Notably, the values of *Tc* were also in reasonable agreement with those found by other authors for PHB biosynthesized by *Bacillus megaterium* [59]. However, the nanocomposites showed lower fusion enthalpy compared to that of PHB and thus a lower crystallinity degree *X_c_*. In this case, the nanoparticles could have slowed and hindered the crystallization, as also reported in lignin/PHB nanocomposites [61]. This may be related to a slight reduction in crystal size and a lower *X_c_*, as a result of the increased number of heterogeneous nucleation sites in the presence of the BaTiO_3_ nanoparticles. When PHB crystallizes in the presence of the nanoparticles, crystals preferably grow on the nanoparticle surfaces, and reductions in overall polymer crystallinity can be expected to occur from the introduction of discontinuities in the matrix crystal structure caused by the sparse growth of crystals at the nanoparticle–matrix interface [62]. 

The presence of a small endothermic peak at 50 °C in all DSC thermograms is typical of PHB. Indeed, some studies have demonstrated that this effect can be ascribed to the presence of an intermediate nanophase between the crystals and the surrounding amorphous material. This nanophase is not crystalline and is formed by the continuation of partially crystallized polymer macromolecules, which straddle the rigid and fluid phases. This is due to the polymer macromolecules, which are much longer than the crystalline nanophase. Therefore, this portion of the material, in which the mobility of the molecules is prevented by the nearby presence of crystalline lamellae, is called “rigid amorphous fraction” (RAF) and possesses a mobility lower than that of the amorphous phase, which, instead, is called “mobile amorphous phase” (MAF). When the material is heated, the molecules constituting the RAF begin to mobilize when temperatures of 30–50 °C are reached, a phenomenon that causes the occurrence of enthalpy recovery preceding melting during heating [34,35]. Furthermore, the presence of multiple peaks in the melting region of PHB was also observed. The literature provides several explanations for this phenomenon, e.g., simultaneous processes of melting–recrystallization, different perfection of crystallites and the presence of two or more crystal forms (i.e., polymorphism) [34,63].

Moreover, it was observed that the polymer stiffness increased with the filler content, and at the same time, it caused a drop in the elongation at break from 5.3% for plain PHB to 2.5% when 5% (w/w%) BaTiO_3_ was added. Therefore, the elongation at break remained almost constant for the different nanocomposites. The reduction with respect to the plain PHB was due to the presence of the nanoceramic inside the polymer matrix, which led to a stress concentration near the filler, thus acting as a source of enhanced nanocomposite brittleness [64]. With the addition of filler up to 15% (w/w%) and 20% (w/w%), the Young’s moduli reached on average 1.9 GPa and 2.0 GPa, respectively, which are 33% higher than that of plain PHB (1.5 MPa). This was caused by the particle stiffening effect of the nanocomposite as the nanoceramic filler content increased. The obtained range of Young’s moduli approached those measured in healthy dry female vertebrae (i.e., 2.16 ± 0.53 GPa) and wet male iliac crest (i.e., 3.03 ± 1.63 GPa), showing potential for some orthopedic and otologic applications [65]. The tensile strength was reduced with increasing filler content. On the one hand, this result could suggest that the filler was not properly acting as a strengthening agent, probably due to the low interfacial adhesion strength between the nanoparticles and the matrix [66]. On the other hand, it should be considered that the mechanical properties of plain PHB decreased with increasing filler content, since the polymer was subjected to increasing melting cycles. Interestingly, the results of the compressive tests highlight that although the compressive strength of the PHB decreased across four melting cycles, the corresponding melting cycles applied to produce the nanocomposites gave rise to averagely increased compressive strengths with increasing BaTiO_3_ content. In this case, although there was none statistically significant difference, the average compressive strengths were always higher than that of the PHB that underwent the same thermal cycles, suggesting that the presence of the ceramic nanoparticles compensated for the weakening of PHB upon repeated melting cycles due to degradation. However, the higher standard deviations observed upon compressive tests, with respect to those obtained upon tensile tests, highlight some shortcomings in terms of repeatability, possibly due to the way the cubic samples were obtained (i.e., blade cutting), which might have hindered the possibility of detecting statistically significant differences among the samples. 

In our study, it was possible to observe a low piezoelectric response of pure PHB (*d*_31_ = 4.15 pm/V; *g*_31_ = 0.113 Vm/N) compared to an enhanced piezoelectric performance of the nanocomposites, which increased with the BaTiO_3_ content. The highest piezoelectric response was thus observed for the nanocomposite with the highest filler content (20 w%), in which *d*_31_ reached 37 pm/V and *g*_31_ 0.67 Vm/N. The inclusion of barium titanate, even at low quantities, in the polymer matrix allowed enhanced piezoelectric properties to be achieved. The enhancement in piezoelectric coefficients induced by an increased content of piezoelectric nanoceramics, such as BaTiO_3_, LiNbO_3_ and ZnO, in piezoelectric polymer-based nanocomposites is in line with other studies [36,67].

The composite brittleness caused some difficulties during the third phase, namely, the 3D printing. Indeed, 3D printing composites for bone scaffolding may be quite challenging because of the rigid materials used, along with the pore size and porosity requirements [23]. We were able to successfully fabricate cube-shaped porous scaffolds. Several scaffolds were 3D printed, with 5/95 (w/w%) BaTiO_3_/PHB being the easiest filament composition to print, and 20/80 (w/w%) BaTiO_3_/PHB representing the most difficult one. The latter showed high brittleness; thus, the ability to precisely tune the printing parameters to improve the 3D printing process was essential. 

The fourth phase focused on the characterization of the 3D printed scaffolds. They showed suitable pore size (600–770 µm range) and porosity (54–62% range), which were lower than their designed values, 1000 µm and 70%, respectively, thus suggesting an overall shrinking effect of the scaffold upon 3D printing [68]. Scaffold biodegradation tests were performed in saline solution for 8 weeks at 37 °C. It is known that PHAs, including PHB and its copolymer PHBV, undergo hydrolytic biodegradation with slow biodegradation kinetics [49,69]. PHBV films have been reported to show very slow hydrolytic degradation *in vitro*, with only 5% mass loss being observed after 8 months in phosphate buffered saline [69]. However, the co-presence of different molecules within PHBV, e.g., as a blend with olive tree leaf extract, even in a low amount, was reported to change the biodegradation profile of this copolymer [70]. In our study, a slow biodegradation rate was observed, with mass losses of 4.5% for plain PHB and 3.3% for 20/80 (w/w%) BaTiO_3_/PHB. Moreover, by taking into account the 20/80 (w/w%) composition of the nanocomposite, it is also possible to argue that the BaTiO_3_ filler promoted the retention of the polymer, thus increasing the stability of the system under the tested conditions to levels acceptable for bone tissue engineering applications. Even though bone tissue may need up to 1 year for its complete regeneration after fracture, depending on the extent of the damage and fracture type, the crucial steps leading to bone healing occur in the first 2–3 months [71]. 

PHB has been investigated for bone tissue engineering due to its good mechanical properties and optimal cytocompatibility [55]. To this end, we aimed at preserving the best biocompatibility of BaTiO_3_/PHB nanocomposites by developing a manufacturing process without any additives. In future research, biocompatible additives or plasticizers could be investigated in the nanocomposite preparation process to avoid multiple melting cycles and to further improve the obtained mechanical properties [72].

Overall, the PHB-based nanocomposite scaffold containing 20% (w%) BaTiO_3_ demonstrated promising features for prospective bone replacement in some applications dealing with spongy or flat bones, such as iliac crest, vertebrae, mastoid bone or sinus lift augmentation, thus possibly meeting several reconstructive needs in orthopedics, otology and dentistry. The 20/80 (w/w%) BaTiO_3_/PHB nanocomposite showed the best stiffness and piezoelectric response among the different compositions tested. However, it was the most brittle one, generating issues in using filament-assisted 3D printing. Considering this, a 3D printer equipped with a pellet cartridge with optimal temperature control could help to overcome the handling and manufacturing limitations experienced with this nanocomposite. Further *in vitro* studies should also be conducted to confirm the cytocompatibility of the nanocomposites and the ability of such piezoelectric scaffolds to promote both osteogenesis and vasculogenesis. Having a mechanically robust biodegradable piezoelectric scaffold able to support the regeneration of vascularized bone tissue could advance bone-tissue-engineered substitutes towards a functional clinical performance. 

## 5. Conclusions

This study was aimed at developing BaTiO_3_/PHB nanocomposites for the 3D printing of bone scaffolds designed for bone tissue engineering. Via mixing and extrusion, we produced PHB-based nanocomposites containing 5%, 10%, 15% and 20% (w%) BaTiO_3_ in the form of filaments, which were successfully 3D printed in porous cubic shapes, showing pore sizes in the 600–770 µm range and porosity in the 54–62% range. The filler was finely dispersed in all the nanocomposites. The thermal and mechanical properties of the bulk samples were evaluated, with the latter demonstrating a stiffening effect rising with BaTiO_3_ concentration, as well as a drop in the elongation at break even with the lowest amount of filler (5% w%). According to our findings, PHB films had moderate piezoelectric properties (*d*_31_ = 4.15 pm/V; *g*_31_ = 0.113 Vm/N); on the other hand, an enhanced piezoelectric performance was achieved in the nanocomposites, which increased with the BaTiO_3_ content. Indeed, the highest piezoelectric response was observed in the 20% (w%) barium titanate nanocomposite (*d*_31_ = 37 pm/V and *g*_31_ = 0.67 Vm/N). Overall, 20/80 (w/w%) BaTiO_3_/PHB displayed the best mechanical and piezoelectric properties; however, its filaments were the most difficult to print. The 20/80 (w/w%) BaTiO_3_/PHB scaffold also showed a 3.3% mass loss in saline solution at 37 °C after 2 months, which indicates it is a long-lasting material in the biological environment. Finally, biological studies of these nanocomposites are expected to possibly disclose their clinical relevance and provide new strategies for vascularized bone tissue engineering. 

## Figures and Tables

**Table 1:** Prepared nanocomposites and identification labels.

BaTiO_3_ Weight Fraction	BaTiO_3_/PHB (w/w%)
0 (plain PHB)	0/100
0.05	5/95
0.10	10/90
0.15	15/85
0.20	20/80

**Table 2:** DSC results reporting the melting and crystallization parameters of PHB (0/100 w/w%) and BaTiO_3_/PHB nanocomposites. Glass transition temperature (*T*_g_) was evaluated via DMTA.

BaTiO_3_/PHB (w/w%)	*T*_m_ (°C)	*T*_g_ (°C)	*X*_c-heat_ (%)	*T*_c_ (°C)	*X*_c-cool_ (%)
0/100	172	12	60	113	60
5/95	172	9	50	111	53
10/90	172	6	51	111	50
15/85	173	6	51	112	51
20/80	173	5	48	111	52

**Table 3:** Compressive strength at 40% strain of plain PHB after different extrusion cycles.

Extrusion Cycle	Compressive Strength (MPa)
0	45.9 ± 5.0
I	38.8 ± 6.0
IV	36.0 ± 2.0

**Table 4:** Dielectric constant (*ε*_d_), converse piezoelectric coefficient (*d*_31_) and direct (*g*_31_) piezoelectric coefficient evaluated for PHB (0/100 w/w%) and BaTiO_3_/PHB nanocomposites.

BaTiO_3_/PHB (w/w%)	*ε*_d_	*d*_31_ (pm/V)	*g*_31_ (Vm/N)
0/100	4.15	4.15	0.113
5/95	4.81	7.03	0.165
10/90	5.00	12.56	0.284
15/85	5.78	18.30	0.358
20/80	6.29	37.46	0.673

**Table 5:** Comparison between designed and experimental pore sizes (mm) and porosities (%) for the 3D printed PHB (0/100 w/w%) and BaTiO_3_/PHB (5/95 w/w% and 20/80 w/w%) nanocomposite scaffolds.

BaTiO_3_/PHB (w/w%)	Pore Size (mm)		Porosity (%)	
	Designed	Experimental	Designed	Experimental
0/100	1.00	0.77	70	62
5/95	1.00	0.60	70	54
20/80	1.00	0.66	70	58

## Data Availability

Data will be made available upon reasonable request to the corresponding author.

## Supplementary Materials

The following supporting information can be downloaded at: https://www.mdpi.com/article/10.3390/bioengineering11020193/s1, Figure S1. Thermal treatment program applied for all DSC measurements reported in present work. Figure S2. Torque vs. time during PHB and BaTiO_3_/PHB nanocomposite mixing. The investigation of torque was carried out in a Brabender mixer in conjunction with the WinMix program, to know the mechanical and thermal effects. Figure S3. BaTiO_3_ nanoparticles: (A) Scanning electron microscopy (SEM) micrographs of the particles as supplied; (B) Nanoparticle frequency (%) vs. diameter range (nm), as detected at the surfaces of the different PHB BaTiO_3_/PHB nanocomposites via SEM analysis, evaluated using ImageJ software. Figure S4. Elemental analysis (EDX) performed on: (A) 5/95 (w/w%) PHB BaTiO_3_/PHB and (B) 10/90 (w/w%) PHB BaTiO_3_/PHB nanocomposites confirmed the presence of BaTiO_3_ nanoparti-cles by the detection of Ba and Ti (circles), and also showed the presence of some impurities, like sodium, potassium, magnesium, aluminum and chlorine (arrows). * indicates the element (i.e., Pt) added for sputter coating the surface. Figure S5. Differential scanning calorimetry (DSC) thermograms of PHB and BaTiO3/PHB nanocomposites: (A) First heating; (B) Cooling; and (C) second heating curves.
