# Supplementary material for: 3D Printed Piezoelectric BaTiO3/Polyhydroxybutyrate Nanocomposite Scaffolds for Bone Tissue Engineering
Source: Bioengineering (Basel). 2024 Feb 17;11(2):193. doi: 10.3390/bioengineering11020193 (PMC10886384; doi:10.3390/bioengineering11020193)
Supplement: Supplementary file 1 [file bioengineering-11-00193-s001.zip › bioengineering-2828507-supplementary.pdf]

bioengineering

MDPI

Supplementary

3D printed piezoelectric $BaTiO_3$/polyhydroxybutyrate nano-composite scaffolds for bone tissue engineering

Giovanna Strangis [1], Massimliano Labardi [2], Giuseppe Gallone [1,*], Mario Milazzo [1], Simone Capaccioli [2,3], Francesca Forli [4], Patrizia Cinelli [1,2], Stefano Berrettini [4], Maurizia Seggiani [1], Serena Danti [1,2,†,*] and Paolo Parchi [5,†]

[1] Dept. of Civil and Industrial Engineering, University of Pisa, Largo L. Lazzarino 2, 56122 Pisa, Italy; giovanna.strangis@phd.unipi.it (G.S.); mario.milazzo@unipi.it (M.M.); patrizia.cinelli@unipi.it (P.C.); maurizia.seggiani@unipi.it (M.S.);
[2] Institute for Chemical and Physical Processes (IPCF), National Researches Council (CNR), Pisa Research Area, via Moruzzi 1, 56124 Pisa, Italy; labardi@df.unipi.it (M.L.);
[3] Dept. of Physics "Enrico Fermi", Largo Pontecorvo 3, 56127 Pisa, Italy; simone.capaccioli@unipi.it (S.C.);
[4] Dept. of Surgical, Medical, Molecular Pathology and Emergency Medicine, University of Pisa, 56126 Pisa, Italy; francesca.forli@unipi.it (F.F.); s.berrettini@med.unipi.it (S.B.);
[5] Dept. of Translational Research and New technologies in Medicine and Surgery, University of Pisa, Pisa, Italy; paolo.parchi@unipi.it (P.P.)
† These authors contributed equally to this work.
* Correspondence: giuseppe.gallone@unipi.it (G.G.); serena.danti@unipi.it (S.D.)

Citation: To be added by editorial staff during production.

Academic Editor: Firstname Last-name

Received: date
Revised: date
Accepted: date
Published: 17 February 2024

Copyright: © 2024 by the authors. Licensee MDPI, Basel, Switzerland. This article is an open access article distributed under the terms and conditions of the Creative Commons Attribution (CC BY) license (https://creativecommons.org/licenses/by/4.0/).

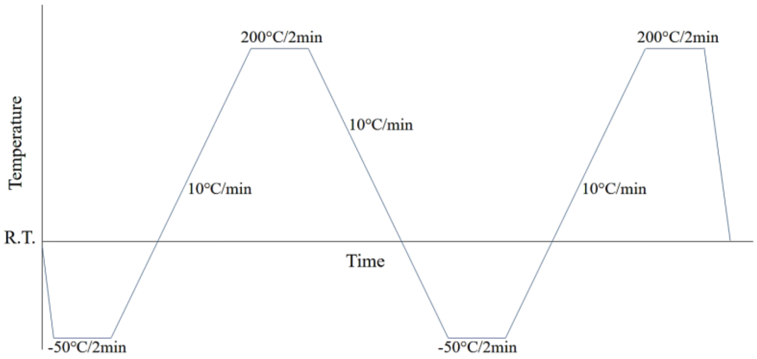

Figure S1. Thermal treatment program applied for all DSC measurements reported in present work.

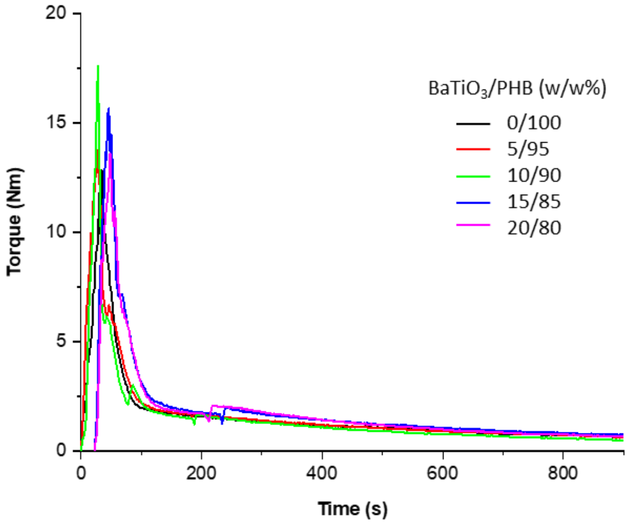

Figure S2. Torque *vs.* time during PHB and $BaTiO_3$/PHB nanocomposite mixing. The investigation of torque was carried out in a Brabender mixer in conjunction with the WinMix program, to know the mechanical and thermal effects.

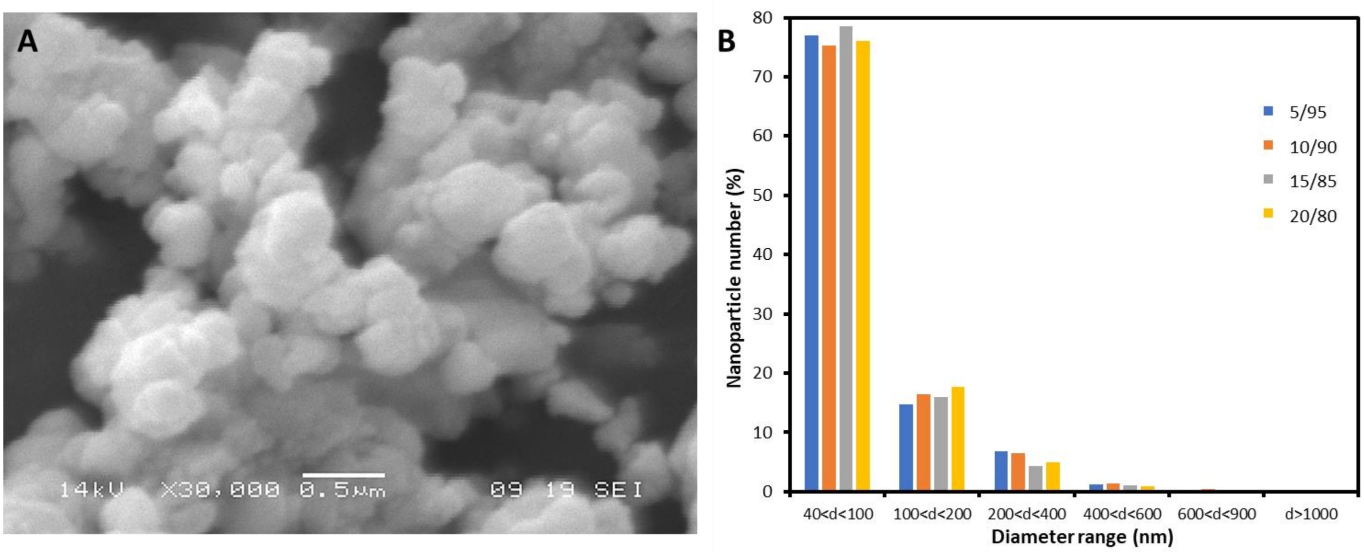

Figure S3. BaTiO3 nanoparticles: (A) Scanning electron microscopy (SEM) micrographs of the particles as supplied; (B) Nanoparticle frequency (%) *vs* diameter range (nm), as detected at the surfaces of the different PHB $BaTiO_3$/PHB nanocomposites via SEM analysis, evaluated using ImageJ software.

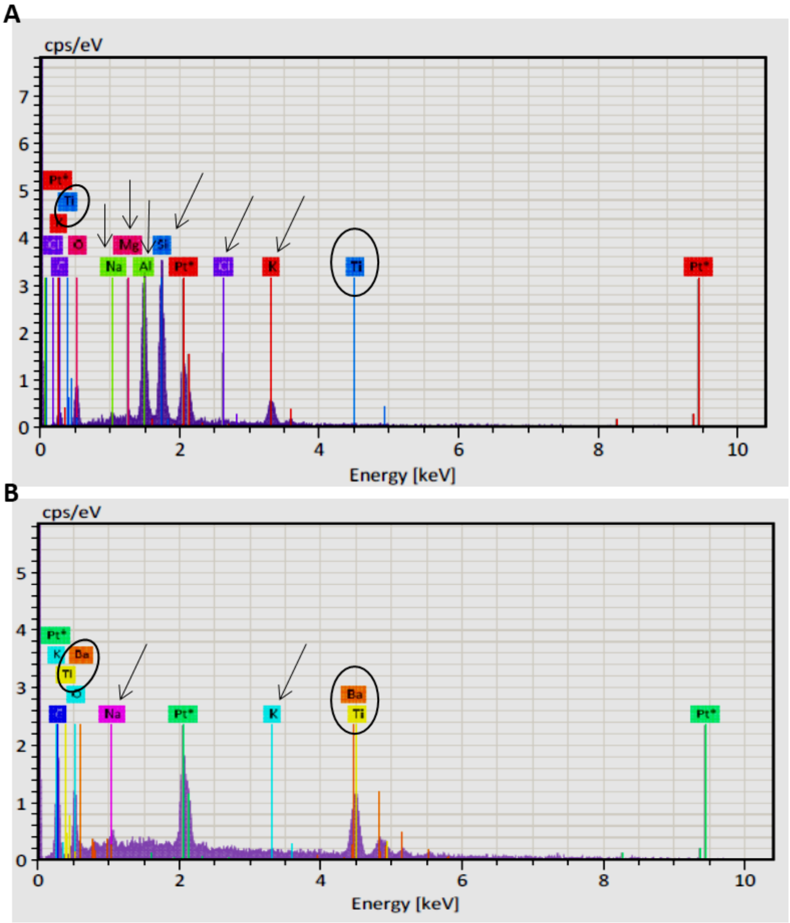

Figure S4. Elemental analysis (EDX) performed on: (A) 5/95 (w/w%) PHB $BaTiO_3$/PHB and 10/90 (w/w%) PHB $BaTiO_3$/PHB nanocomposites confirmed the presence of $BaTiO_3$ nanoparticles by the detection of Ba and Ti (circles), and also showed the presence of some impurities, like sodium, potassium, magnesium, aluminum and chlorine (arrows). * indicates the element (i.e., Pt) added for sputter coating the surface.

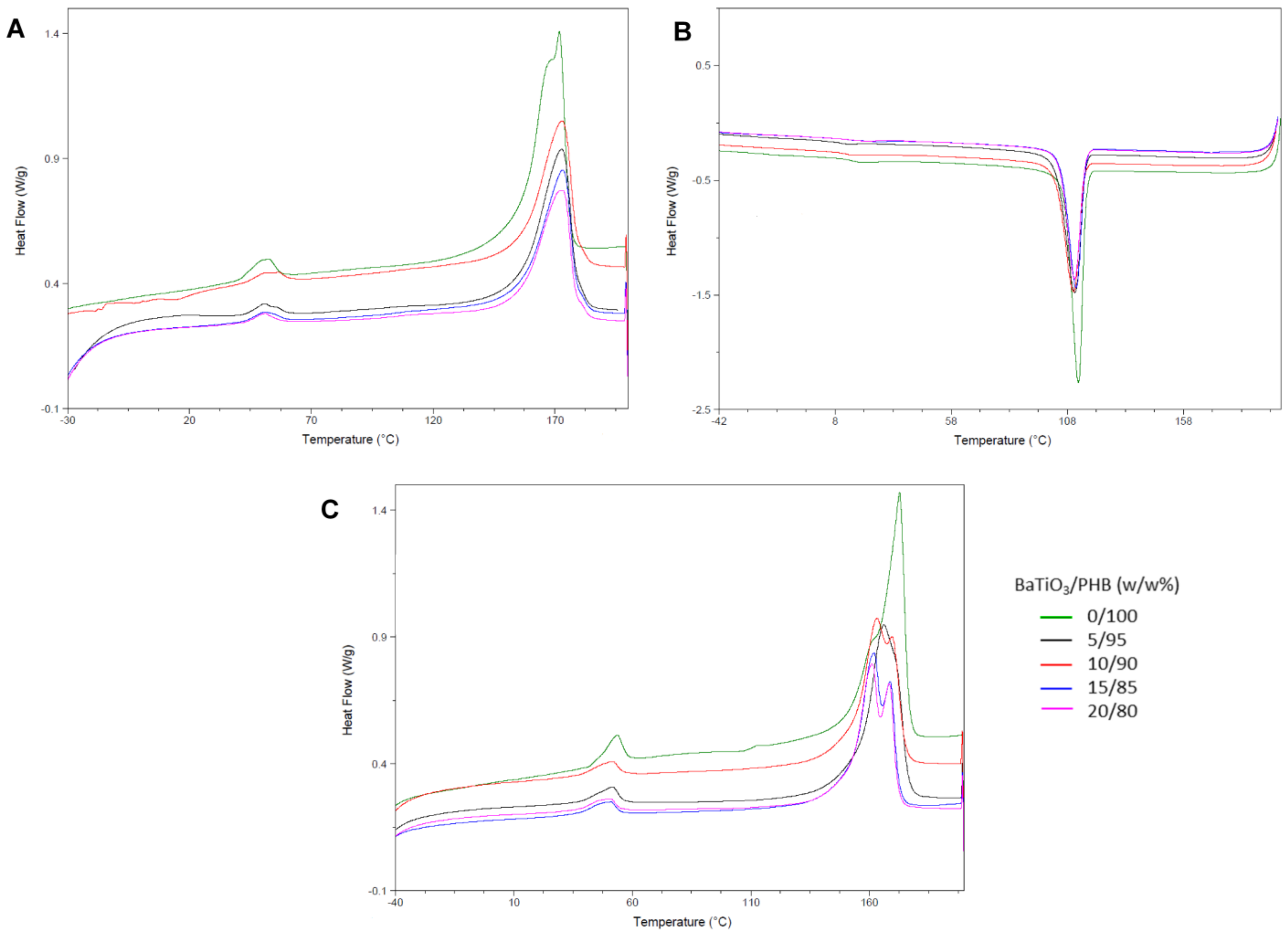

Figure S5. Differential scanning calorimetry (DSC) thermograms of PHB and $BaTiO_3$/PHB nanocomposites: (A) First heating; (B) Cooling; and (C) second heating curves.

Disclaimer/Publisher's Note: The statements, opinions and data contained in all publications are solely those of the individual author(s) and contributor(s) and not of MDPI and/or the editor(s). MDPI and/or the editor(s) disclaim responsibility for any injury to people or property resulting from any ideas, methods, instructions or products referred to in the content.
